# Supplementary material for: Interrupting the intergenerational cycle of violence: protocol for a three-generational longitudinal mixed-methods study in South Africa (INTERRUPT_VIOLENCE)
Source: BMC Public Health. 2023 Feb 27;23:395. doi: 10.1186/s12889-023-15168-y (PMC9969039; doi:10.1186/s12889-023-15168-y)
Supplement: Supplementary file 2 — Additional file 2: Supplement 2. Young Carers 3 – Caregiver Questionnaire. [file 12889_2023_15168_MOESM2_ESM.pdf]

Supplement 2: Young Carers 3 – Caregiver Questionnaire

ABOUT YOU	
Demographic information	Age, home language and basic demographic will be asked using four items [1], South Africa General Household Survey [2], and the South Africa Demographic and Health Survey [3]. Questions on sexuality, gender and sex assigned at birth were modeled on the US Consortium of Higher Education LGBT Resource Professionals Self-Study [4].
Education	Highest level of education attained will be measured using items from the IMAGES Study South Africa 2018 [5].<br><br>Additional items will focus on completed schooling: school dropout and reasons for drop out and whether they accessed tertiary education.<br><br>Violence exposure at school was measured with one item.
Your status	5 items will ask about their citizenship status, whether they are a South African citizen, if no, which country, if they have a birth certificate, ID book or passport and if no, why they don't have.
**YOUR HOME**	
Your home	5 items will identify whether the caregiver lives with the young adult and the type of accommodation they currently live in vs the type of accommodation they occupied 10 years ago at study follow-up.<br><br>21 items will be used to identify size of the home, type of home, number of people living in it and the relationships to the participants based on items from the South African Census [1] and IMAGES Study [5]. These cover if house is owned or rented, number of rooms, kitchen, toilets, number of chronically ill people in the home, number of employed people, whether adults and children share beds, the household water and electricity source and how many days either were not available in the past month.
Employment	Six items from the IMAGES Study South Africa [6] assess employment and household income focusing on employment of respondent, whether the work is seasonal, part-time or full-time, the monthly household income, number of adults and children dependent on the household income.
Making ends meet	Food insecurity will be measured using the three item Household Hunger Scale (HHS). This is a subscale of the HFIAS (Household Food Insecurity Scale). It consists of 3 items and has demonstrated the potential for cross-cultural validity. The HFIAS has been validated throughout different countries, including South Africa [7]. One additional item is included to assess whether children had enough food in the past 4 weeks.<br><br>8 items from necessities approach of Barnes and Wright [8] and 6 additional items will measure what necessities the household was able to afford in the past month. These will include three meals a day, sanitary products, household cleaning products, electricity, water, shoes amongst others.

	4-items from the Sinovuyo Teen Study will assess a household's financial management including membership in e.g. a stokvel or burial society, borrowing money and ability to cope with financial shocks [9].
Government grants	Receipt within the household of the major forms of social security transfers (Child Support Grant, Foster Child Grant, Care Dependency Grant, Disability Grant, Grant in Aid, government housing subsidy, Old Age Pension) will be measured. An additional item will ask if the household received the special COVID-19 Social Relief of Distress grant and the one time R700 grant provided in 2020 as a result of the COVID-19 pandemic.
Communicating with others	Two items will assess whether households have access to a mobile phone or smart phone, TV, radio and computer/internet and whether and what type of health messaging they may have received through their devices in the past year.
**YOUR FAMILY AND FRIENDS**	
Social Support	The 8-item Modified Medical Outcome Study Social Support Survey (mMOS-SS) will be used to measure social support [10] which has similarly excellent validity compared to the original 18-item MOS-SS. The MOS-SS was designed to measure social support received by persons with chronic conditions. It has good internal consistency α=0.91to 0.97 and test-retest reliability α=0.72 to 0.78 [11]. It has been used in our previous studies in South Africa with good success [12,13].
**YOUR CHILDREN**	
Your relationship with (name of adult participant)	9 items will be asked to assess the relationship between the participants and the young adult participating in the study, whether the young adult lived with the participant for the whole duration of her/his childhood, if the young adult is biological, whether she/he breastfed, if the participants still financially supporting the young adult and how it was to care for the young adult.
Disciplining and Protecting (name of young adult participant)	The ISPCAN Child Abuse Screening Tool – ICAST-P (parent self-report) will be used to on the use of physical and emotional punishment and the young adults' experience of neglect, sexual abuse, domestic violence and sibling bullying. Items on positive discipline are also included [14]. This will be adapted to allow retrospective reporting of abuse when caregivers were parenting the young adult during their adolescence.
Illness in early childhood (young adult)	One item will assess whether the caregiver had to take the young adult to a hospital during their childhood for illnesses such as pneumonia, HIV, malnutrition, burns, convulsions, diarrhoea or injuries.
Current children you are caring for	14 items will assess whether the caregiver currently has a child in their care, their relationship to that child and the roles of caregiving they carry out. These will also assess whether the caregiver had children that have died and the causes they have died of.

Pregnancy	Two items for women assess the number of times they have been pregnant and whether the pregnancy for the last child they are caring for was planned.
Child protection	Two items will assess whether the child are currently caring for was exclusively breastfed for 6 months, and whether their birth was registered
Fathering section	For men, one item will assess whether the pregnancy had been planned.
Parenting stress	Parenting stress will be assessed using the 18-item Parenting Stress Scale (PSS) which was developed as an alternative to the 101-item Parenting Stress Index [15]. It provides a measure that considers positive aspects of parenting as well as the negative, focusing on traditional “stressful” aspects. The PSS has previously been used in a longitudinal cohort study in South Africa [16].
Children’s education	Nine items will assess whether the index child attends crèche or school, whether they receive a fee exemption, receive free school books, school meals, school trips, school uniform or school transport.
Early childhood parenting	38 items from the Comprehensive Early Childhood Parenting Questionnaire (CECPAQ) support, structure and stimulation sub-scales will be used to assess engagement with the index child, creating play opportunities for the index child, disciplining the child and consistency of parenting behaviours [17]. This measure has not been previously used in South Africa but was piloted extensively with some cultural adaptations e.g. toys and games specific to South Africa.<br><br>7 items from Involved Parenting subscale of the Alabama Parenting Questionnaire [18] measured parental involvement in the index child’s activity. These have been previously used in South Africa with good validity and reliability [12].
Attitudes to corporal punishment	Three self-developed items assessed attitudes towards disciplining children. These cover endorsement of physical discipline, gendered discipline and parenting similarly vs differently to one’s own parents.
Child abuse and neglect – Disciplining child	39 items from the ISPCAN Child Abuse Screening Tool parent self-report (ICAST-P) will be used to self-report on the use of physical and emotional punishment and the child’ experience of neglect, sexual abuse, domestic violence and sibling bullying. Items on positive discipline are also included [14].
The health of your children - oldest child in your care	Seven items covering injuries and burns, diarrhea and vomiting, pneumonia or bronchitis, skin conditions, general body pains, cold/flu and COVID-19 will be retained from the previous questionnaire. Pulmonary TB will be measured using the TB symptom assessment used in the baseline and follow-up research. Access to health care will be assessed with 8 items from the REACH study [19].<br><br>Disability will be assessed using 9 items from the Short Set on Functioning Washington Group Questionnaire (WG-SS) [20]. These cover visual, auditory, mobility and cognitive disabilities.
HIV - oldest child in your care	9 items adapted from Sinovuyo Teen Study (2015) will be asked to assess if their children have been tested for HIV, if yes, are they positive, if yes, are they taking any medication, did they need to go to hospital in the last month,

	did they tell their child that they are HIV-positive, do they know their child's CD4 and if they can tell us [21]
**YOUR RELATIONSHIPS**	
Your relationships when (adult participant) was growing up	Participants' relationship status and experiences will be measured using items from the IMAGES Study South Africa 2018 [5]. Items will assess current partners and partners 10 years ago when the adolescent lived with them. The items will assess what their main motivation in staying in the relationship if they have one, if their partners drink alcohol, use drugs or have HIV, how many one night stands they had in the past year and if any of their sexual partners were from the same sex. These items also assess the nature of the relationships ranging from main partners to casual partners and side partners.
Relationship Beliefs (male & female version)	The Gender Equitable Men (GEM) Scale measures attitudes toward gender norms in intimate relationships or differing social expectations for men and women [22]. 8 items from the Inequitable scale will be used and 2 additional items specific to the South African context on lobola and rape were added. Each item is scored on a 3-point scale, where 1 = agree, 2 = partially agree, and 3 = do not agree. The estimate of internal consistency = .81, but it is expected to vary according to the setting. This measures has been previously used in South Africa [23,24].
Relationship control scale (male & female version)	The Sexual Relationship Power Scale (SRPS) measures power within sexual relationships for both men and women. 8 items of the Relationship Control subscale are used in the questionnaire. Items are scored on a 4-point Likert scale, with 1 = strongly agree, 2 = agree, 3 = disagree, and 4 = strongly disagree) [25].
Intimate Partner Violence	IPV perpetration will be measured using the 19-item questionnaire from the UN Multi-country cross-sectional study on men and violence and Asia and the Pacific [26,27]. Questions will ask if the behaviour happened in the past 12 months, if yes, the frequency in the last year and the frequency in the woman's lifetime.<br><br>IPV experience will be measured using the 25 item WHO domestic violence instrument from the WHO Multi-Country Study on Violence Against Women [28] for past year and lifetime exposure. Five additional items assess IPV experience in pregnancy. For women, additional questions cover whether they have sought help, whom they have sought help from and what the response to their disclosure was.
Community Violence	8 items from WHO community violence perpetration instrument developed by Tremblay et al., [29] and adapted by Jewkes & Morrell [30] will be used to assess whether a participant has taken part in violent activities within the community in which they live .
Risky sex	Items from the National survey of HIV and risk behaviour amongst young South Africans [31] will be retained to measure sexual activity (screening item), age of sexual debut, frequency of condom use, sex with an older partner, sexual activity under the influence of alcohol and under the influence of drugs, and

	self-perceived risk of HIV. Items from this survey were also used to measure forced sex (by verbal and physical force) and pregnancy (14 items).<br><br>Women's transactional sex with men will be measured using a measure from a larger study on gender-based violence and HIV infection in Soweto [32]. Men's transactional sex with women who are not their wife or main partner will be assessed using the 25 item female version adapted for men [33] .
Non-partner sexual violence	Non-partner sexual violence against males and females perpetrated by men will be measured using 5 items from the UN Multi-country Study on men and violence [27]. There will be follow-up questions after the items such as how old they were when the incident first happened, who did it, how old they were when the last happened.
**MY COMMUNITY**	
Community Violence	Lifetime and past-year community violence exposure (witnessing, experiencing) and its frequency will be measured using 14 items from the Social and Health Assessment (SAHA; Exposure to Violence Scales [34]with 5-point response options ranging from 'none' (0) to 'ten or more times' (4). The internal consistency of the scale in the US study is found to be good (α = .78 and α = .89).
Community Participation	Three items from the adapted Critical Consciousness Scale [35] will assess community participation with regards to how much participant agree that community members would intervene if they saw a fight, a child skipping school, or someone selling drugs on the street.
Community Cohesion	Six items adapted from the Stepping Stones Questionnaire [36] will assess community cohesion with a specific focus on safety e.g. walking around at night. A 4-point likert-scale will assess participant's agreement with the statement of how safe the community feels.
Additional Trauma	10 items are included from The Harvard Trauma Questionnaire (HTQ) which is a checklist written by the Harvard Program in Refugee Trauma to assess trauma and torture [37]. Among the items, there are imprisonment, torture, unnatural death, kidnapping.
**YOUR HEALTH**	
Depression Symptoms	The Patient Health Questionnaire (PHQ-9) is a nine item self-report screening tool for accessing the severity of depression based on DSM-IV criteria and scored on a 4-point scale where 0 = not at all, 1 = several days, 2 = more than half of the days and 3 = nearly every day over the last two weeks [38]. This has been previously used in South Africa [39].
Disability	The 6-item Washington Group Short Set (WG-SS) on Functioning will be used to assess disability status [40]. This is a comprehensive measure to determine disability in six domains of functioning: seeing, hearing, walking/climbing steps, remembering/concentrating, self-care, and communication. Each item is scored on a 4-point scale where 0 = no difficulty, 1 = some difficulty, 2 = a lot

	of difficulty, and 3 = cannot do at all. The WG-SS has been extensively tested and validated and used in over 80 countries [40].
Anxiety	The General Anxiety Disorder (GAD)-7 seven item questionnaire measures symptoms of GAD following DSMV-IV symptom criteria for GAD and assigns scores of 0, 1, 2, and 3, to the response categories of 'not at all', 'several days', 'more than half the days', and 'nearly every day', respectively. Using the threshold score of 10, the GAD-7 has a sensitivity of 89% and a specificity of 82% for GAD [41]. This tool and its 2-item form have been used in South Africa [42,43]
Suicide Screening Questions	5 items are from the National Institute for Mental Health's Ask Suicide Screening Questions (ASQ) Toolkit [44]. It was developed as a brief, straight-forward tool to identify potential suicide risk. It includes items like 'in the past few weeks, have you wished you were dead' and 'have you ever tried to kill yourself'.
Chronic conditions / illnesses	HIV status will be measured using the Verbal Autopsy Questionnaire which is a checklist of AIDS-defining illnesses and AIDS-non-specific illnesses. The Verbal Autopsy uses an algorithm of AIDS-defining illnesses to determine HIV status [45] with a specificity and sensitivity above 74% [46].<br><br>A voluntary oral HIV-self test will also be offered to participants. These have shown to have 99% sensitivity and 100% specificity [47] as well as high acceptability among users [48].<br><br>Chronic illnesses common in South Africa such as asthma, HIV, diabetes, hypertension, low blood pressure and epilepsy will be measured based on clinical diagnosis.
Trauma	The PTSD-8 is a short self-report instrument to screen for post-traumatic stress disorder [49]. This includes four intrusive thoughts items, two avoidance items and two hypervigilance items. These are answered on a four-point likert scale ('not at all' – 'all the time). This has not been previously used in South Africa.
Alcohol Use	The 3-item Alcohol Use Disorders Identification Test Consumption (AUDIT-C) was developed as a simple method of screening for excessive drinking **[50]**. This measures frequency of drinking, number of units typically drunk and excessive consumption.
Substance Use	5 items from the Alcohol, Smoking and Substance Involvement Screening Test (ASSIST) will be used to assess substance use **[51]**. These cover use of tobacco products, cannabis, inhalants, nyaope and other drugs not prescribed by a doctor. Its validity has been studied in the South African context, yielding good results **[52]**.
Accessing health services	10 Items from the REACH study – a 4-province study of health care utilization amongst HIV+ people, will be used to measure health care utilization [19], traditional healers, the church or religious leaders. An additional open item will allow report of reasons for non-access to healthcare (this should allow estimation of accessibility, affordability and acceptability – including affordability of transport.

	4 additional items on access and uptake of contraception have been added for women.
Help when you need it	7 items will measure if the participant has a community health worker, and/or social worker and/or a religious leader and/or a traditional healer who is supporting them when they need help. If the participant yes to either one of them, a further question will ask how and in what way they support the participant.
Psychotic experiences	6-items adapted from the Community Assessment of Psychic Experiences (CAPE) will be used [53]. This is a self-report measure for assessing phenomena that are similar to the positive symptoms of psychosis [54,55]. Each item is scored on a 4-point Likert scale, where 1= never, 2= sometimes, 3= often, 4= nearly always.
**YOUR CHILDHOOD**	
Your childhood<br><br>Child abuse and neglect	Physical, emotional and sexual child abuse exposure will be measured using the ISPCAN Child Abuse Screening Tool retrospective version [56], ICAST-R. Two additional items will measure exposure to domestic violence in the home. The tool includes four sub-scales on physical, emotional and sexual abuse and neglect with follow-up questions on when and how often this happened, which people did this and how much this experience hurt/harm them.<br><br>Access to services and reactions to abuse disclosure will be measured using adapted items from the ISPCAN Child Abuse Screening Tool – child self-report version ICAST-C [57] and WHO Multi-Country Study on Violence Against Women [28]. Questions will measure disclosure, who this disclosure was made to, whether it was supported or unsupported and any service receipt following disclosure.
Family conflict	2 items will assess whether there was a lot of arguing and shouting between adults at home and whether the adults at home hit each other before the participant turned 18.
Adverse life events	18 items from the Adverse Childhood Experiences International Questionnaire (ACE-IQ) will measure parental morbidity, parental mortality, parental substance use, parental educational neglect, parental imprisonment, parental abandonment, violent events within the community, police violence and foster care experience [58] which has been previously used successfully with adolescents in Malawi [59].
Peer victimization	Bullying victimization and perpetration was measured using the 12-item Brief Zurich Bullying scales (6-items victimization, 6 items perpetration) covering instances of verbal, physical and exclusionary bullying as well as sexual and homophobic events [60].
**OVERCOMING CHALLENGES**	
Self-esteem	The Rosenberg Self-Esteem Scale [61] is a widely used 10-item self-report measure for assessing individual's both negative (e.g., 'I certainly feel useless

	at times') and positive feelings (e.g.,'On the whole I am satisfied with myself') about themselves and their self-worth. It's a short and easy to administer scale. The items are answered using a 4-point Likert scale from strongly agree to strongly disagree.
Resilience	The 12 adapted items from the Child and Youth Resilience Measure (CYRM-12), 5-point version were used to assess socio-ecological resilience among participants [62,63].
**COVID-19**	
COVID-10 Isolation	7 self-developed items will ask about the participants' experience of lockdown, whom they were in lockdown and how many people, if they were in lockdown with an essential worker, if they were in lockdown at their current place and if they had access to a garden or an outside area.
Childcare	Two items will ask if COVID-19 had affected their employment and who cared for their children if they had to go to work.
Occupational Impact (Employment)	11 items will assess whether, due to COVID-19, any of the participants' household members lost their jobs, whether any of them went to bed hungry during lockdown, if anyone in the household died, if there was more violence in the household than usual, and how their mental health was during lockdown.

References

1 Statistics South Africa. South AFrican Census Questionnaire 2011. 2011.

2 Statistics South Africa. General Household Survey South Africa 2019. 2020.

3 Statistics South Africa. South Africa Demographic and Health Survey 2016. 2019.

4 Consortium of Higher Education LGBT Resource Professionals. Consortium of Higher Education LGBT Resource Professionals 2018 Self-Study Report. 2018.

5 Promundo. IMAGES - International Men and Gender Equality Study. 2018.

6 Promundo. IMAGES - International Men and Gender Equality Study. 2018.

7 Deitchler M, Ballard T, Swindale A, *et al.* Validation of a Measure of Household Hunger for Cross-Cultural Use. Washington, DC: 2010.

8 Barnes H, Wright G. Defining child poverty in South Africa using the socially perceived necessities approach'. In: Minujin A, Nandy S, eds. *Global Child Poverty and Well-Being: Measurement, Concepts, Policy and Action*. Bristol: : Policy Press 2012. 135–54.

9 Steinert JI, Cluver LD, Meinck F, *et al.* Household economic strengthening through financial and psychosocial programming: Evidence from a field experiment in South Africa. *J Dev Econ* 2018;**134**:443–66. doi:10.1016/J.JDEVECO.2018.06.016

10 Moser A, Stuck AE, Silliman RA, *et al.* The eight-item modified Medical Outcomes Study Social Support Survey: psychometric evaluation showed excellent performance. *J Clin Epidemiol* 2012;**65**:1107. doi:10.1016/J.JCLINEPI.2012.04.007

11 Sherbourne C, Stewart A. The Medical Outcomes Survey (MOS) social support survey. *Soc Sci Med* 1991;**32**:705–14.

12 Cluver L, Meinck F, Steinert J, *et al.* Parenting for Lifelong Health: A pragmatic cluster randomised controlled trial of a non-commcialised parenting programme for adolescents and their families in South Africa. *BMJ Glob Health* 2018.

13 Casale M, Wild L, Cluver L, *et al.* Social support as a protective factor for depression among women caring for children in HIV-endemic South Africa. *J Behav Med* 2015;**38**:17–27. doi:10.1007/s10865-014-9556-7

14 Runyan D, Dunne MP, Zolotor AJ, *et al.* The development and piloting of the ISPCAN Child Abuse Screening Tool-Parent version (ICAST-P). *Child Abuse Negl* 2009;**33**:826–32. doi:10.1016/j.chiabu.2009.09.006

15 Berry JO, Jones WH. The parental stress scale: Initial psychometric evidence. *J Soc Pers Relatsh 1995 123 P 463-472* 1995;**12**:463–72.

16 Rochat TJ, Houle B, Stein A, *et al.* Psychological morbidity and parenting stress in mothers of primary school children by timing of acquisition of HIV infection: a longitudinal cohort study in rural South Africa. *J Dev Orig Health Dis* 2018;**9**:41–57. doi:10.1017/S204017441700068X

17 Verhoeven M, Deković M, Bodden D, *et al.* Development and initial validation of the comprehensive early childhood parenting questionnaire (CECPAQ) for parents of 1–4 year-olds. *Eur J Dev Psychol* 2017;**14**:233–47. doi:10.1080/17405629.2016.1182017

18 Elgar FJ, Waschbusch DA, Dadds MR, *et al.* Development and validation of a short form of the Alabama Parenting Questionnaire. *J Child Fam Stud* 2007;**16**:243–59.

19 Schneider H, McIntyre D. Researching Equity in Access to Health Care (REACH) - Final Technical Report. Johannesburg: 2012.

20 The Washington Group. WG Short Set on Functioning (WG-SS). 2020.https://www.washingtongroup-disability.com/question-sets/wg-short-set-on-functioning-wg-ss/ (accessed 15 Jun 2021).

21 Cluver L, Lachman J, Ward C, *et al.* Developing a parenting programme to prevent child abuse in South Africa: A pre-post pilot study. *Res Soc Work Pract* Published Online First: 2016. doi:0.1177/1049731516628647

22 Pulerwitz J, Barker G. Measuring Attitudes toward Gender Norms among Young Men in Brazil. *Men Masculinities* 2008;**10**:322–38. doi:10.1177/1097184X06298778

23 Gottert A, Barrington C, Pettifor A, *et al.* Measuring Men's Gender Norms and Gender Role Conflict/Stress in a High HIV-Prevalence South African Setting. *AIDS Behav* 2016;**20**:1785–95. doi:10.1007/s10461-016-1374-1

24 Jewkes R, Sikweyiya Y, Morrell R, *et al.* Gender Inequitable Masculinity and Sexual Entitlement in Rape Perpetration South Africa: Findings of a Cross-Sectional Study. *PLoS ONE* 2011;**6**:e29590. doi:10.1371/journal.pone.0029590

25 Pulerwitz J, Gortmaker SL, DeJong W. Measuring Sexual Relationship Power in HIV/STD Research. *Sex Roles* 2000;**42**:637–60. doi:10.1023/A:1007051506972

26 Fulu E, Jewkes R, Roselli T, *et al.* Prevalence of and factors associated with male perpetration of intimate partner violence: findings from the UN Multi-country Cross-sectional Study on Men and Violence in Asia and the Pacific. *Lancet Glob Health* 2013;**1**:e187-207. doi:10.1016/S2214-109X(13)70074-3

27 Fulu E, Miedema S, Roselli T, *et al.* Pathways between childhood trauma, intimate partner violence, and harsh parenting: findings from the UN Multi-country Study on Men and Violence in Asia and the Pacific. *Lancet Glob Health* 2017;**5**:512–22. doi:10.1016/S2214-109X(17)30103-1

28 Garcia-Moreno C, Jansen H, Ellsberg M, *et al.* WHO Multi-country Study on Women's Health and Domestic Violence against Women - Initial results on prevalence, health outcomes and women's responses. Geneva: : WHO 2005.

29 Tremblay RE, Pagani-Kurtz L, Mâsse LC, *et al.* A bimodal preventive intervention for disruptive kindergarten boys: its impact through mid-adolescence. *J Consult Clin Psychol* 1995;**63**:560–8. doi:10.1037//0022-006x.63.4.560

30 Jewkes, R, Morrell, R. Hegemonic Masculinity, Violence, and Gender Equality: Using Latent Class Analysis to Investigate the Origins and Correlates of Differences between Men. *Men Masculinities* 2018;**21**.https://journals.sagepub.com/doi/abs/10.1177/1097184X17696171 (accessed 26 Nov 2022).

31 Reproductive Health Research Unit. HIV and sexual behaviour among young South Africans: A national survey of 15-24 year olds. Parklands: : loveLife 2005.

32 Dunkle K, Jewkes R, Brown H, *et al.* Transactional sex among women in Soweto, South Africa: prevalence, risk factors and association with HIV infection. *Soc Sci Med* 2004;**59**:1581–92.

33 Medical Research Council. War at Home: Preliminary findings of the Gauteng Gender Violence Prevalence Study. Johannesburg: : Medical Research Council 2010.

34 Ruchkin V, Vermeiren R, Schwab-Stone M. *The Social and Health Assessment (SAHA): Psychometric developmental summary*. New Haven: : Yale University 2004.

35 Diemer MA, McWhirter EH, Ozer EJ, *et al.* Advances in the Conceptualization and Measurement of Critical Consciousness. *Urban Rev* 2015;**47**:809–23. doi:10.1007/s11256-015-0336-7

36 Jewkes R, Nduna M, Levin J, *et al.* Impact of stepping stones on incidence of HIV and HSV-2 and sexual behaviour in rural South Africa: cluster randomised controlled trial. *BMJ* 2008;**337**:a506. doi:10.1136/bmj.a506

37 Mollica RF, Caspi-Yavin Y, Bollini P, *et al.* The Harvard Trauma Questionnaire. Validating a cross-cultural instrument for measuring torture, trauma, and posttraumatic stress disorder in Indochinese refugees. *J Nerv Ment Dis* 1992;**180**:111–6.

38 Adewuya AO, Ola BA, Afolabi OO. Validity of the patient health questionnaire (PHQ-9) as a screening tool for depression amongst Nigerian university students. *J Affect Disord* 2006;**96**:89–93. doi:10.1016/j.jad.2006.05.021

39 Bhana A, Rathod SD, Selohilwe O, *et al.* The validity of the Patient Health Questionnaire for screening depression in chronic care patients in primary health care in South Africa. *BMC Psychiatry* 2015;**15**:118. doi:10.1186/s12888-015-0503-0

40 Washington Group on Disability Statistics. An Introduction to the Washington Group on Disability Statistics Question Sets. 2020.

41 Spitzer RL, Kroenke K, Williams JBW, *et al.* A Brief Measure for Assessing Generalized Anxiety Disorder. *Arch Intern Med* 2006;**166**:1092. doi:10.1001/archinte.166.10.1092

42 Henn C, Morgan B. Differential item functioning of the CESD-R and GAD-& in African and white working adults. *SA J Ind Psychol* 2019;**45**:10.

43 van Heyningen T, Honikman S, Tomlinson M, *et al.* Comparison of mental health screening tools for detecting antenatal depression and anxiety disorders in South African women. *PLOS ONE* 2018;**13**:e0193697. doi:10.1371/journal.pone.0193697

44 Horowitz LM, Bridge JA, Teach SJ, *et al.* Ask suicide-screening questions (ASQ): A brief instrument for the pediatric emergency department. *Arch Pediatr Adolesc Med* 2012;**166**:1170–6. doi:10.1001/archpediatrics.2012.1276

45 Lopman B, Barnabas R, Boerma T, *et al.* Creating and validating an algorithm to measure AIDS mortality in the adult population using verbal autopsy. *Public Libr Sci Med* 2006;**3**:e312.

46 Lopman B, Cook A, Smith J, *et al.* Verbal autopsy can consistently measure AIDS mortality: a validation study in Tanzania and Zimbabwe. *J Epidemiol Community Health* 2010;**64**:330–4. doi:10.1136/jech.2008.081554

47 Devillé W, Tempelman H. Feasibility and robustness of an oral HIV self-test in a rural community in South-Africa: An observational diagnostic study. *PLOS ONE* 2019;**14**:e0215353. doi:10.1371/journal.pone.0215353

48 Hector J, Davies M-A, Dekker-Boersema J, *et al.* Acceptability and performance of a directly assisted oral HIV self-testing intervention in adolescents in rural Mozambique. *PloS One* 2018;**13**:e0195391. doi:10.1371/journal.pone.0195391

49 Hansen M, Andersen TE, Armour C, *et al.* PTSD-8: A Short PTSD Inventory. *Clin Pract Epidemiol Ment Health* 2010;**1**:101–8. doi:10.2174/1745017901006010101

50 Saunders JB, Aasland OG, Babor TF, *et al.* Development of the Alcohol-Use Disorders Identification Test (Audit) - Who Collaborative Project on Early Detection of Persons with Harmful Alcohol-Consumption .2. *Addiction* 1993;**88**:791–804. doi:10.1111/j.1360-0443.1993.tb02093.x

51 WHO ASSIST Working Group. The Alcohol, Smoking and Substance Involvement Screening Test (ASSIST): development, reliability and feasibility. *Addiction* 2002;**97**:1183–94.

52 van der Westhuizen C, Wyatt G, Williams JK, *et al.* Validation of the Alcohol, Smoking and Substance Involvement Screening Test in a low- and middle-income country cross-sectional emergency centre study. *Drug Alcohol Rev* 2016;**35**:702–9. doi:10.1111/dar.12424

53 Aloba O, Opakunle T. The Brief 10-Item Community Assessment of Psychic Experiences-Positive Scale (Brief CAPE-P10): Initial psychometric properties, gender measurement invariance and mean differences among Nigerian adolescents. *Early Interv Psychiatry* 2020;**14**:723–33. doi:10.1111/EIP.12903

54 Ribeaud D, Murray A, Shanahan L, *et al.* Cohort Profile: The Zurich Project on the Social Development from Childhood to Adulthood (z-proso). *J Dev Life-Course Criminol* 2022;**8**:151–71. doi:10.1007/s40865-022-00195-x

55 Shanahan L, Steinhoff A, Bechtiger L, *et al.* Frequent teenage cannabis use: Prevalence across adolescence and associations with young adult psychopathology and functional well-being in an urban cohort. *Drug Alcohol Depend* 2021;**228**:109063. doi:10.1016/j.drugalcdep.2021.109063

56 Dunne MP, Zolotor AJ, Runyan DK, *et al.* ISPCAN Child Abuse Screening Tools Retrospective version (ICAST-R): Delphi study and field testing in seven countries. *Child Abuse Negl* 2009;**33**:826–32. doi:10.1016/j.chiabu.2009.09.005

57 Zolotor AJ, Runyan DK, Dunne MP, *et al.* ISPCAN Child Abuse Screening Tool Children's Version (ICAST-C): Instrument development and multi-national pilot testing. *Child Abuse Negl* 2009;**33**:833–41. doi:http://dx.doi.org/10.1016/j.chiabu.2009.09.004

58 World Health Organization. Adverse Childhood Experiences International Questionnaire (ACE-IQ) - Rationale for ACE-IQ. Geneva: : WHO 2012. http://www.who.int/violence_injury_prevention/violence/activities/adverse_childhood_experiences/en/

59 Kidman R, Smith D, Piccolo LR, *et al.* Psychometric evaluation of the Adverse Childhood Experience International Questionnaire (ACE-IQ) in Malawian adolescents. *Child Abuse Negl* 2019;**92**:139–45. doi:10.1016/J.CHIABU.2019.03.015

60 Murray AL, Eisner M, Ribeaud D, *et al.* Validation of a Brief Self-Report Measure of Adolescent Bullying Perpetration and Victimization. *Assessment* 2021;**28**:128–40. doi:10.1177/1073191119858406

61 Rosenberg M. Society and the adolescent self-image. *Princet NJ Princet Univeristy Press* 1965.

62 Liebenberg L, Ungar M, LeBlanc JC. The CYRM-12: a brief measure of resilience. *Can J Public Health Rev Can Sante Publique* 2013;**104**. doi:10.1007/BF03405676

63 Ungar M, Liebenberg L. Assessing Resilience Across Cultures Using Mixed Methods: Construction of the Child and Youth Resilience Measure. *J Mix Methods Res* 2011;**5**:126–49. doi:10.1177/1558689811400607
